# Some Masters of Mediæval Surgery

**Published:** 1918-01-19

**Authors:** 


### GLIMPSES BACKWARD.*

### II. Some Masters of Mediæval Surgery.

It is not unnatural, perhaps, to regard surgery, in all but its most primitive sense, as a purely modern product, and to consider antisepsis as entirely the outcome of modern investigation and research. Like so many of our up-to-date theories, a great part, if not the whole, of Lister's work had been anticipated by the surgeons of five and six centuries ago—imperfectly, it is true, for they had not then the aid of the microscope; but in a manner nevertheless sufficiently striking.

### The School of Alexandria.

Surgery in some form or another has been practised from time immemorial. The exigences of combat and the hazards of the chase led to certain men among all primitive people possessing or claiming to possess special skill in the staunching of blood, the extraction of arrows, binding up of wounds, setting broken limbs, and the like. Amongst the Hindoos and Chinese elaborate treatises on medicine and surgery were in existence at the time of Alexander, and much of the proverbial wisdom of the East filtered westward, and had its influence upon the great medical teachers at Alexandria.

Here, in the days of the Ptolemys (*circa* 300 B.C.), for space does not allow us to dwell upon the extraordinary advances made by Hippocrates, the study of anatomy flourished, and vivisection was performed upon criminals given for that purpose by the king. Lithotomy was practised by specialists, the operation differing but little from that described in Buddhist writings of much earlier date, and an instrument was devised for crushing stones within the bladder. One, Herophilus, gained especial renown for his researches upon the brain (the *torcular Herophili* was first described by him, as was the *calamus scriptorius*), and boldly operated upon internal organs such as the liver and spleen; the latter he considered "of little consequence in the animal economy." Later writers, such as Galen and Celsus, reflect and perpetuate the teachings of the Alexandrian school. The latter's celebrated account of lithotomy is that of the operation as there practised, and he is also the first to give a detailed description of amputation. Many famous surgeons flourished throughout the period of the Empire; but on the fall of Rome their knowledge was in great part lost to the world, or hidden away in some retired monastery, where it remained till rediscovered by the scholars of the Renaissance.

During the dark period which succeeded, the Arabians were regarded as the great exponents of medicine and surgery. But their religion forbade the practice of anatomy, as it induced them to endure their sufferings with equanimity, and it is not till the foundation of the school of Salerno that surgery was practised with any real understanding.

### The North Italian Surgeons.

From thence the centre of interest travels northwards, and a brilliant group of surgeons, Bruno, Hugo of Lucca and his son Theodoric, and, greatest of all, William of Salicet and his pupil Lanfranchi. It is so usual to consider anæsthesia as having been invented in the middle of the nineteenth century that it is surprising to find Hugo and his son putting their patients to sleep with opium and mandragora, as well as with an inhalant mixture, the composition of which is not exactly known. Yet some such insensibility to pain must have been produced from very early times, or the operations surgeons performed would have been impossible.

The most generally used antiseptic was wine. Thus Bruno recommends that in penetrating wounds of the abdominal cavity, if difficulty was encountered in replacing the intestines, they should be pressed back with a sponge soaked in warm wine. If the omentum found its way out of the wound, all that was black or green was to be cut off. If the intestines were injured, the opening should be sutured with fine silk thread, great care being taken to effect a complete closure of the wound.

Both Hugo of Lucca and his son were surgeons at Bologna, and they also laid great stress upon the value of wine as a dressing for wounds. Theodoric, indeed, boasts of his scars, "*pulcherrimas cicatrices*," so small as to be scarcely noticeable, and brought about by a scrupulous cleansing of the wound surfaces, and the avoidance of any powders or ointments. The expression "union by first intention" comes to us from the olden time, nor could it have been used save by men who had solved the problems of antisepsis in a practical, if not a theoretical, manner.

Bruno's pupil and the master of Lanfranchi, William of Salicet, was yet another *alumnus* of the great Bolognan faculty. He was in many ways an original man, quoting but little from his predecessors, and then only if his own observations had verified their statements. He was an expert ophthalmologist, as well as an aurist and general surgeon. Nasal and aural polypi were to be either excised or their stalk ligatured and the stump allowed to shrivel. Ranula was treated by the cyst being lifted well forward by means of a sharp iron hook, and then being split with a razor and the calculus removed. The tendency to reform was recognised, and it was recommended that the cavity was to be filled with vitriol powder (? copper sulphate) or alum and salt, so as to ensure closure by means of adhesive inflammation.

(*Continued on opposite page*—334.)

* The previous article appeared on January 5, p. 285.

### Glimpses Backward

(*continued from opposite page*—335).

LANFRANCHI IN PARIS.

His pupil Lanfranchi, after practising as physician and surgeon in Milan till banished from there for political reasons about 1290, fled first to Lyons, where his brilliant work attracted so much attention that the dean of the medical faculty in Paris invited him to fill the chair of surgery at the new university there. Here the renown of his teaching and his lectures at the bedside attracted an incredible number of students. So great, indeed, was the eagerness to be present at his operations that all could not be accommodated, and the dean urged him to embody his methods in a book. This appeared in 1296, dedicated to Philippe le Bel, then King of France. This work had an extraordinary reputation throughout Europe, and together with Guy de Chauliac's equally celebrated treatise was the standard text-book till the days of Paré. Among much other excellent teaching he advocates the suture of severed nerves, deprecated by previous writers, holding that the paralysed limb regained its functions more rapidly if this be done.

GUY DE CHAULIAC.

Lanfranchi had transferred pre-eminence in surgery from Italy to France, and though scholars still went to the older universities for post-graduate work, as they did for several centuries after, interest now chiefly centres around the schools of Paris and Montpelier.

Mondeville, the contemporary of Lanfranchi, seems to have been one of the first to introduce a systematic study of anatomy into the curriculum. A man of philosophical temperament and wide erudition, his advice to young physicians on the choice of a nurse and the limitations of his art recall that of Abernethy to a later generation. By the meddlesomeness of those not qualified to effect a cure "it often happens that diseases in themselves curable grow to be simply incurable or are made much worse than they were before."

In passing from Mondeville to his great pupil Guy de Chauliac, we seem to approach very near to the spirit of our own times. Born about 1370, of presumably humble parents, in the town whose name he bears, he qualified at Montpelier, and thence proceeded to make an extended tour of the Italian universities, staying for long at Bologna under the famous anatomist Bertruccio. At Montpelier he had among his teachers a Scotchman, Bernard Gordon, whilst a famous Englishman, John of Gaddesden, the first royal physician—mentioned, incidentally, by Chaucer in his "Doctor of Physic"—was a fellow-student. Dissection was freely practised at Bologna, rather contradicting the general belief that it was prohibited by the Popes, as we learn from Guy's great work the "Chirurgia Magna." "My master, Bertruccius," he says, "conducted the dissection very often after the following manner: having placed the dead body on a bench, he used to make four lessons upon it. In the first, the nutritional portions were treated, because they are the first to become putrefied. In the second, he demonstrated the spiritual members; in the third, the animate members; in the fourth, the extremities." Again: "In the bodies of men, of apes, and of pigs and of many other animals, tissues should be studied by dissections, and not by pictures, as did Henricus (Mondeville), who was seen to demonstrate anatomy with thirteen pictures." Whilst his aphorism that "the surgeon ignorant of anatomy carves the human body as a blind man carves wood" has become famous.

Chauliac's book is confessedly a compilation. He took all that was good in the methods of his own time and added thereto "whatever his own measure of intelligence enabled him to find useful." The great cavities, the skull, the thorax, and abdomen, were freely opened by him, though in injuries of the former he insists that unless specially contra-indicated, expectant treatment was to be the rule. He has seen, he says, severe injuries of the cranium accompanied by loss of brain substance be followed by complete recovery of the patient and little impairment of function. He recognised that wounds of the intestines were surely fatal unless leakage could be prevented. He lays down exact indications for the opening of the thorax, then, even as now, the *bête noire* of surgery, pointing out the relations of the ribs and diaphragm, so as to show by what incisions fluid could safely be removed. It is to him that we owe a method of taxis for the reduction of hernia in use till the nineteenth century, though a radical operation, in which the testicle was removed and the canal obliterated by the cautery or corrosive agents, was also practised.

His was a bold and fearless spirit, not afraid to reproach his predecessors in that "they follow one another like cranes, whether for love or fear, I cannot say." In Guy de Chauliac we find the first awakening of the Renaissance spirit that was to end the long darkness of the Middle Age.

